# Fixation positions after skipping saccades: A single space makes a large difference

**DOI:** 10.3758/s13414-012-0365-1

**Published:** 2012-09-21

**Authors:** André Krügel, Françoise Vitu, Ralf Engbert

**Affiliations:** 1 Department Psychologie, Universität Potsdam, Karl-Liebknecht-Str. 24/25, 14476 Potsdam, OT Golm Germany; 2 Laboratoire de Psychologie Cognitive, CNRS, Aix-Marseille Université, Centre St Charles, 3 Place Victor Hugo, 13331 Marseille, France

**Keywords:** Eye movements, Reading, Motor control, Skipping

## Abstract

During reading, saccadic eye movements are generated to shift words into the center of the visual field for lexical processing. Recently, Krügel and Engbert (Vision Research 50:1532–1539, 2010) demonstrated that within-word fixation positions are largely shifted to the left after skipped words. However, explanations of the origin of this effect cannot be drawn from normal reading data alone. Here we show that the large effect of skipped words on the distribution of within-word fixation positions is primarily based on rather subtle differences in the low-level visual information acquired before saccades. Using arrangements of “x” letter strings, we reproduced the effect of skipped character strings in a highly controlled single-saccade task. Our results demonstrate that the effect of skipped words in reading is the signature of a general visuomotor phenomenon. Moreover, our findings extend beyond the scope of the widely accepted range-error model, which posits that within-word fixation positions in reading depend solely on the distances of target words. We expect that our results will provide critical boundary conditions for the development of visuomotor models of saccade planning during reading.

**Electronic supplementary material:**

The online version of this article (doi:10.3758/s13414-012-0365-1) contains supplementary material, which is available to authorized users.

When we read a line of text, our eyes initially skip over up to one-third of all words during the first pass (Rayner, 1998). Much attention has been paid to the factors that influence a reader’s decision to skip the next word. Independent effects of the length, the predictability, and the frequency of the next word have been identified as important for the cognitive decision to trigger word skipping (Brysbaert, Drieghe, & Vitu, 2005; Drieghe, Desmet, & Brysbaert, 2007; Rayner & McConkie, 1976; Rayner, Sereno, & Raney, 1996; Rayner, Slattery, Drieghe, & Liversedge, 2011; Rayner & Well, 1996; Vitu, O’Regan, Inhoff, & Topolski, 1995). However, word length turned out to be the most important variable determining word skipping: Short words are more frequently skipped than long words (and so are high-predictability words and high-frequency words when word length is controlled for). Here we focus on the analysis of average landing positions after normal (i.e., from word *N* to word *N* + 1) and skipping (from word *N* to word *N* + 2) saccades.

Where the eyes land within words is primarily determined by low-level visuomotor variables such as interword spaces or the distances and lengths of target words (Rayner, 1998). Most importantly, average landing positions of saccades vary systematically as a function of the prior distance of the eyes (i.e., the launch-site distance) from the target word (McConkie, Kerr, Reddix, & Zola, 1988). Each one-letter increment of the launch-site distance shifts the distribution of subsequent fixation locations within the next word about half a letter to the left. This well-established finding is often interpreted as a signature of a saccadic range error (Kapoula, 1985) during reading (McConkie et al., 1988). As a result, average first-fixation positions at word centers without systematic under- or overshoot (McConkie et al., 1988; O’Regan, 1981; O’Regan & Lévy-Schoen, 1987; Rayner et al., 1996; Vitu, O’Regan, & Mittau, 1990) are realized only from specific launch-site distances.

In a recent study, Krügel and Engbert (2010) demonstrated that word skipping is another important factor that influences saccade landing positions during reading (see also Radach, 1996; Radach & Kempe, 1993; Radach & McConkie, 1998). Using a large corpus of eye-movement data, Krügel and Engbert ran separate analyses for normal and skipping saccades for identical launch-site distances. As a result, landing positions could be decomposed by saccade types. Word skipping strongly modulated the launch-site effect by inducing a large additional leftward shift of the average initial fixation position within the target words. By such an extra leftward displacement of two or more letters (depending on the launch-site distance), the effect of skipped words turned out to be as large as the effect of an approximately six- or seven-letter increment of launch-site distance.

The present study was motivated by two questions related to the work by Krügel and Engbert (2010). First, it is unclear whether the relocation of saccadic endpoints in word skipping reflects a strategic effect under top-down control or whether it is due to low-level visuomotor constraints. Radach and McConkie (1998) hypothesized that in some cases, readers might aim at an intermediate position between the skipped word and the word after the skipped word so as to keep the skipped word in close foveal distance for further word processing (see also Radach, 1996). If this is the main cause of the effect of word skipping on landing positions in reading, we might expect that the effect would be limited to normal reading conditions. On the other hand, if the effect is a signature of a general visuomotor phenomenon, it should also be present under conditions in which a final eye position near the skipped word provides no processing benefits.

Second, in reading it is difficult to know which word is the target of a given saccade. It is evident from overlapping within-word landing-position distributions that a substantial proportion (about 15 %–20 %) of all saccades miss their target words and result in mislocated fixations on unintended word neighbors (Engbert & Nuthmann, 2008; McConkie et al., 1988). As a consequence, Krügel and Engbert’s analyses were based on advanced statistical techniques (Engbert & Nuthmann, 2008; see also Nuthmann, Engbert, & Kliegl, 2005) used to estimate unbiased probability distributions for landing positions within the target words of intended skipping saccades and intended normal saccades. Such a procedure can be avoided in single-saccade paradigms.

Therefore, we developed a single-saccade task with clear target words in both skipping and normal saccades and with tight experimental control of launch-site distance, word length, and the size of the skipped word. We used meaningless arrangements of “x” letter strings to eliminate lexical processing of the skipped word in order to establish the potentially visuomotor nature of the effect.

## Method

### Participants

A total of 30 students at the University of Potsdam (22 female, 8 male), between 19 and 44 years of age, received study credit or a total of €21 for participating in three 45- to 60-min sessions; they were all naïve with respect to the purpose of the experiment. All of the participants reported normal or corrected-to-normal vision.

### Apparatus

With their heads supported on a chinrest, participants were seated at a viewing distance of 60 cm in front of a 22-in. FT/LCD monitor (refresh rate 60 Hz, resolution 1,680 × 1,050 pixels). The stimuli were presented in fixed-width Courier font with a size of 18 points on the vertical center line of the computer display. Eye movements were recorded binocularly using an EyeLink II system (SR Research, Osgoode/Ontario, Canada) with a sampling rate of 500 Hz and a spatial resolution better than 0.01°.

## Material

**Fig. 1:**
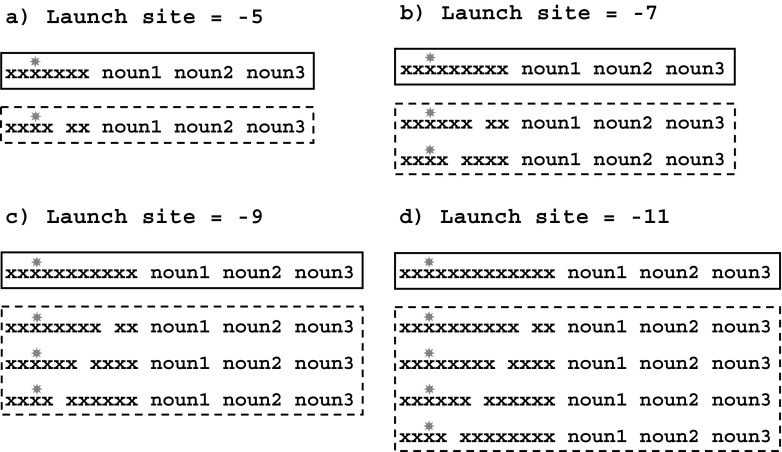
Visual configuration of the stimulus materials. A group of three nouns follows an arrangement of “x” letter strings. The presence or absence of the space in the “x” letter configuration distinguishes skipping saccades (*dashed boxes*) from normal saccades (*solid boxes*) to noun1

The stimulus display consisted of two groups of items: an arrangement of “x” letter strings of variable lengths, followed by three German nouns. Drawn randomly from a pool of 3,888 different nouns, with equal numbers of high- and low-frequency words, 1,296 unique word triplets were generated separately for each participant. These were split equally into three subsets of 432 triplets, defined by the length of the first word. The first noun was a four-, six-, or eight-letter word, the second was a seven-letter word, and the third word had nine, seven, or five letters, respectively, based on the first-word length (all three word lengths summed up to 20 letters). On 48 randomly selected triplets in each subset, one word was replaced by an animal name of the same length, resulting in a total of 144 positive animal-name trials (approximately 11 % of all trials). The position of this animal name within the group of three words was balanced across all selected triplets. The “x” letter string was presented so that the participants’ initial fixation positions were always located at the third “x.” From this starting position, the string of “x”s extended 4, 6, 8 or 10 letters to the right, resulting in varying initial launch-site distances of the eyes to the space before the first noun of –5, –7, –9, or –11 character positions, respectively.^1^ In 50 % of the trials, the foveal string of “x” letters was split into two parts by replacing one of the “x”s by a space; thus, the initial saccade to the first, target word of the triplet required the skipping of the second part of the string. Nested within four conditions of different launch-site distances, the space within the “x” letter string appeared with equal probabilities at one to four different positions, leading to up to four different lengths of the second part of the “x” letter string (see Fig. 1, dashed boxes). In 50 % of the skipping saccade trials, the second part of the “x” letter string started with an uppercase “x.” All factors were counterbalanced within and across the three experimental sessions and were presented in random order.

### Procedure and design

For each participant, the experiment was composed of three sessions that were conducted on three different days. At the beginning of each session, the participants were introduced to the task in a 12-trial practice block; actual testing occurred in four subsequent test blocks with 108 trials per block. Participants were instructed to read a list of three German nouns to determine whether one of the words was the name of an animal and to respond by keypress without making any errors. They were further told to ignore the string(s) of “x” letters and to move their eyes directly to the first word of the triplet. Each trial began with the presentation of a “Ready” signal centrally on a plain white screen, which was replaced after 1 s by a fixation cross at the left of the screen. Both the offset of the fixation cross and the simultaneous onset of the stimulus presentation were then triggered by the participants’ fixations in a predefined area around the fixation cross. The stimulus display remained in view until a response key was pressed. Participants received auditory feedback after each trial (low tone = correct, high tone = incorrect). Finally, all participants were informed about their total performance after every block.

### Data selection

Initial saccades after stimulus onset were analyzed, and trials with eye blinks were excluded from the analyses.

## Results

**Fig. 2:**
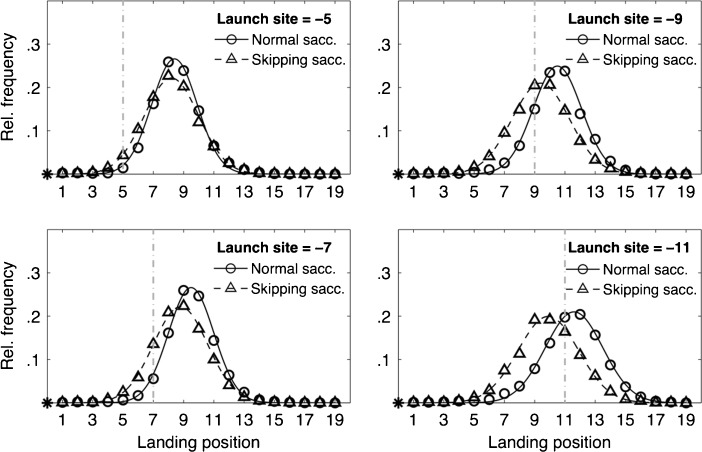
Distributions of landing positions in normal, one-word saccades (*circles*, *solid lines*) and in skipping saccades (*triangles, dashed lines*) for launch-site distances of –5, –7, –9, and –11 letters to the left of the space before the first noun. The asterisks at the origins of the horizontal lines denote saccades’ launch sites, and the numbers on the horizontal line indicate letter positions to the right of the saccades’ launch sites. The vertical dashed gray lines mark the positions of the space before of the first letter of the target words

We were interested in systematic shifts of saccade landing positions for normal, one-word saccades versus skipping saccades. Figure 2 presents comparisons of the overall landing-site distributions observed after normal saccades and skipping saccades for launch-site distances of –5, –7, –9, and –11 letters from the left boundary of the target word.^2^ All distributions of first-fixation positions were fitted by normal distributions (i.e., the lines in Fig. 2). The effect of saccade type (normal vs. skipping) turned out to be significant (paired-sample *t* test comparisons of the mean landing sites in the four different launch-site conditions all demonstrated *p* < .001). Therefore, we reproduced the main effect of normal versus skipping saccades on the distribution of saccade landing sites (Krügel & Engbert, 2010) in a highly controlled single-saccade task.

We did not obtain reliable differences in landing-site distributions for different lengths of the target words, which is different from findings in normal reading (e.g., McConkie et al., 1988). Separate repeated measures one-way analyses of variance (ANOVAs) with Word Length as the factor of variation were carried out for all combinations of launch-site distance and saccade type. In three out of eight tests (launch sites –7 and –9 in simple saccades and launch site –7 in skipping saccades), we found significant effects of word length, with *p* < .01. However, these effects refer to actual differences of the mean landing positions with a maximum of less than 0.3 character spaces. Thus, we used aggregated data across target-word lengths for all further analyses.

**Fig. 3:**
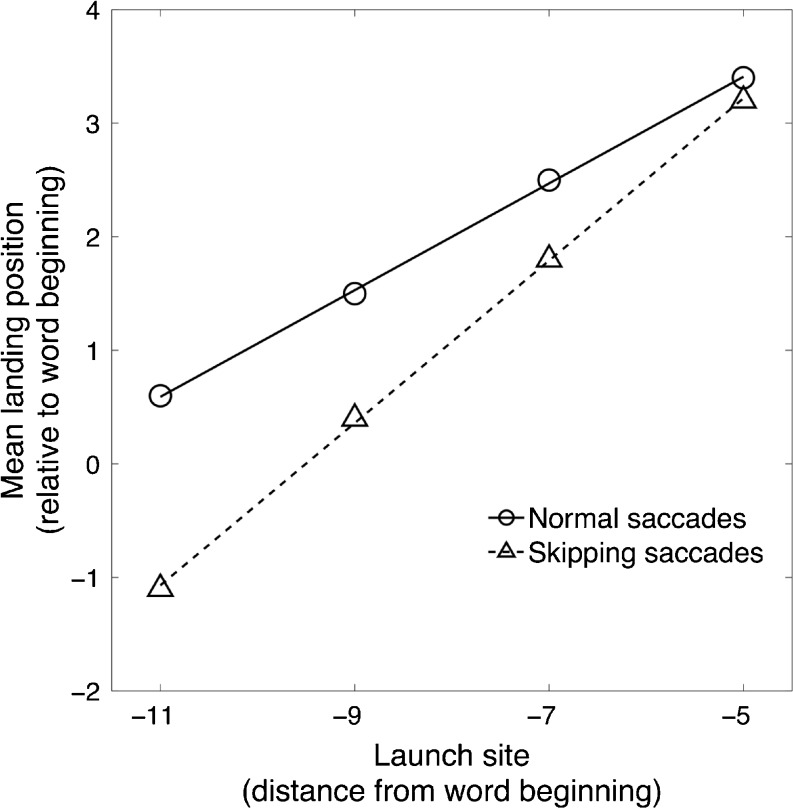
Initial mean landing positions as a function of launch-site distances from word beginning for simple saccades (*circles*, *solid line*) and skipping saccades (*triangles*, *dashed line*). Linear regressions indicate pronounced difference in both slope and intercept of the relations for the two conditions

Next, we investigated systematic interactions between the main factors—that is, Launch-Site Distance and Saccade Type. Figure 3 presents the estimated means of the landing-site distributions and the associated linear regression lines for normal saccades (circles, solid line) and skipping saccades (squares, dashed line) as a function of launch-site distances. A repeated measures two-way ANOVA demonstrated large main effects of launch-site distance [*F*(3, 36862) = 2,475.81, *p* < .001] and saccade type [*F*(1, 36862) = 2,229.99, *p* < .001], as well as a significant interaction [*F*(3, 36862) = 164.58, *p* < .001]. With increasing launch-site distance, mean landing positions were systematically shifted by factors of 0.5 letters in simple saccades and 0.7 letters in skipping saccades toward the beginning of the target words. It is obvious from Fig. 3 that the effect of skipped “x” letter strings turned out to be marginal for the shortest launch-site condition but increased up to a leftward displacement of 1.8 letters as compared to simple saccades for launch-site distances of –11 letters. On the basis of the skipping of meaningless word-shaped objects, these findings qualitatively replicate the main results reported by Krügel and Engbert (2010) for normal reading. Further analyses on the effects of the position of the space within the “x” letter string are provided as supplemental information. Taken together, we concluded that the landing-position effect after skipped words is not restricted to normal reading, but might represent a robust visuomotor phenomenon.

## Discussion

The primary goal of the present study was to test the effect of word skipping on fixation position under highly controlled constraints in a single-saccade paradigm. Using arrangements of “x” letter strings, which were placed before a task-relevant group of three words, we qualitatively replicated the substantial left shift of landing positions after skipping saccades demonstrated by Krügel and Engbert (2010) for normal reading. In particular, we found a strong interaction between the two main effects of launch-site distance and saccade type (i.e., normal vs. skipping saccades).

On a quantitative level, we obtained some differences as compared to the findings of Krügel and Engbert (2010). First, the systematic launch-site-contingent shift of mean landing sites in the present experiment appeared to be larger than in natural reading for both normal and skipping saccades. In contrast to the present estimated regression slopes of 0.53 (normal saccades) and 0.72 (skipping saccades), Krügel and Engbert reported estimates of 0.27 for normal saccades and 0.48 for skipping saccades for normal reading. Second, with a maximum effect size of 1.8 letters, the effect of skipped letter strings appeared to be smaller in the present experiment than in normal reading. Furthermore, we found little effect of the length of the target word. In a recent work, Engbert and Krügel (2010) demonstrated that readers use task-specific prior knowledge about the probability distribution of target distances for optimal target localization on the basis of Bayesian saccade planning. According to such a model, the restricted range of particularly long target distances in the present experiment might have created the quantitative differences in comparison to reading. The range of the long target word distances used in the present experiment may also explain why we did not observe effects of the length of the target words, since the positions of the right boundaries of the target words in the present experiment most frequently fell outside the perceptual span of approximately 14–15 letters to the right of the current fixation position (DenBuurman, Boersma & Gerrissen, 1981; McConkie & Rayner, 1975; Rayner, 1986). Thus, visual information about the lengths of the target words was nearly absent in most of the trials.

Multiple studies on saccadic eye movements have indicated that the distance between saccades’ launch sites and target words systematically influences the mean fixation location in words during reading (McConkie et al., 1988; Nuthmann et al., 2005; Rayner et al., 1996; Reilly & O’Regan, 1998). The demonstration that this effect varies for normal versus skipping saccades is very important, because this phenomenon cannot be explained by current models of saccade generation. Both range error (Kapoula, 1985; McConkie et al., 1988) and Bayesian estimation of the target position (Engbert & Krügel, 2010) are based on launch-site distance and word length as unique predictors of the within-word landing position. The effect may potentially be accounted for by an alternative model based on the global effect (Deubel, Wolf, & Hauske, 1984; Findlay, 1982; Vitu, 2008), although this is still not certain, as this model has not been elaborated to deliver quantitative predictions. A combination of multiple oculomotor mechanisms (range error, global effect, or Bayesian estimation of target positions) working in parallel is also possible. Thus, the present results are challenging to future modeling attempts. Since the presence of an additional single space in skipping saccades and its position relative to the beginning of the target word (see Supplementary Fig. S1) makes a large difference on saccades’ landing sites, new oculomotor models will need to include explicit representations of the positions of spaces within the reading material in addition to other well-known low-level determinants, such as target-word distance and target-word length.

Our results contradict an earlier hypothesis that the shift of the eye’s landing sites toward the beginning of words in word-skipping saccades might occasionally reflect top-down control with the function of keeping the skipped word at a close foveal distance for further word processing after the skipping saccade (Radach, 1996; Radach & McConkie, 1998). In the present experiment, the final eye position in skipping saccades was relocated near the task-irrelevant skipped string of “x”s; indeed, participants were asked to identify animal names within the group of three words to the right of the “x” letter arrangement. Thus, the observed effect of skipped letter strings indicates automatic oculomotor mechanisms. However, our results do not exclude that this automatic, low-level oculomotor mechanism may serve further linguistic processing of the skipped word in normal reading.

Implications of the effect of skipping saccades on fixation position are important for theoretical models of eye guidance in reading. To account for the distribution of fixation positions within words, current models of eye-movement control in reading (e.g., Engbert, Nuthmann, Richter, & Kliegl, 2005; Reichle, Rayner, & Pollatsek, 1999) typically incorporate the launch-site-contingent mean shift of saccadic landing positions within words on the basis of McConkie et al.’s (1988) quantitative estimates. However, the new observation of the effect of word skipping uncovers that McConkie et al.’s (1988) important findings are strongly biased because they mix up two more fundamental populations of saccades—namely, normal and skipping saccades. In effect, current reading models largely overestimate the accuracy of skipping saccades and, even more importantly, underestimate the accuracy of simple progressive saccades to words *N* + 1.

In conclusion, we believe that our results set important boundary conditions for the development of visuomotor models of saccade planning during reading and, at a more general level, for computational models of eye-movement control.

## Electronic supplementary material

Below is the link to the electronic supplementary material.

ESM 1 (PDF 446 kb)

## Footnotes

1 Note that we did not test for launch-site distances of -4 letters to the left of the beginning of the target word which is the minimally required launch-site distance to skip over two-letter words in spaced texts. Two letter words are the shortest words in German.

2 Estimates of landing-site distributions in natural reading are typically based on observations that are truncated at word boundaries (see Engbert & Nuthmann, 2008, for a technical discussion). The results reported here would still be valid if all calculations were carried out using Gaussian fits of landing-position distributions from data restricted to landing positions within the target words.
