# Supplementary material for: Fixation positions after skipping saccades: A single space makes a large difference
Source: Atten Percept Psychophys. 2012 Sep 21;74(8):1556–61. doi: 10.3758/s13414-012-0365-1 (PMC3514692; doi:10.3758/s13414-012-0365-1)
Supplement: Supplementary file 1 — (PDF 446 kb) [file 13414_2012_365_MOESM1_ESM.pdf]

Supplementary Information

Krügel*, A., Vitu, F., & Engbert, R. (2012). Fixation positions after skipping saccades: A single space makes a large difference. Attention, Perception, & Psychophysics.
*e-mail: kruegel@uni-potsdam.de

When normal saccades (i.e., from word N to N+1) and skipping saccades (i.e., from word N to N+2) are launched from equal distances toward target words, the space at the end of the current word N constitutes the most obvious low-level visual difference. In normal one-word saccades, this space is always located one character position in front of the target word; hence, the launch-site word N constitutes a continuous letter string up to the beginning of the target word. In skipping saccades, however, the space at the end of word N is located before the intermediate and skipped word N+1, and therefore interrupts the string of letters between the current fixation position and the beginning of the target word at varying positions, depending on the length of the skipped word N+1. Here we tested how saccadic landing sites depend on the position of the space at the end of the foveal "x" letter string (i.e., the launch-site string).

In Figure S1, saccades' mean landing positions relative to the beginnings of target words are plotted as a function of the position of the space after the launch-site "x" letter string. Position zero on both the horizontal and vertical axes denotes the position of the space before the target word. Positive numbers refer to the letters within the target word, and negative numbers on both axes reflect character positions to the left of the space before the target word. Different symbols and curves separate the data contingent on the different launch-site distances within the experiment. The vertical differences across these curves reflect the main effect of the four launch-site distances realized within the present experiment.

Interestingly, saccadic landing sites vary systematically as a function of the position of the space at the end of the launch-site string. Normal saccades (data points at the 0 horizontal location) are instances in which the space is located immediately before the first letter of the target word; their mean landing positions are farther into the word than are the mean landing positions of skipping saccades. In fact, when the space moves away from the target word and the length of the intermediate letter string increases, landing sites systematically shift in the corresponding leftward direction, leading to a very gradual transition from landing positions in simple saccades to landing positions in skipping saccades. Still, this trend comes to a stop, or even reverses, when the space at the end of the launch-site string approaches the current fixation position and the intervening word is very long.

Thus, the position of the first space to the right of the current fixation position turns out to be an important determinant of saccadic end points and may play a dominant role in the process of saccade planning. In general, this finding is highly compatible with an observation by Pollatsek and Rayner (1982), who demonstrated that the first space to the right of the current fixation "is the primary space information used by readers of English" (Rayner & Pollatsek, 1996, p. 463). In their study, the reading rates of English texts were slowed by 40% to 60% if space information was removed. However, when only the space between words N and words N+1 was preserved, the reading rate recovered to a level of 90% of the reading rate in ordinary spaced texts.

As the effect of the position of the first space to the right of the current fixation position probably underlies the effect of word skipping on initial landing sites in words, it will be worth investigating this further in future studies.

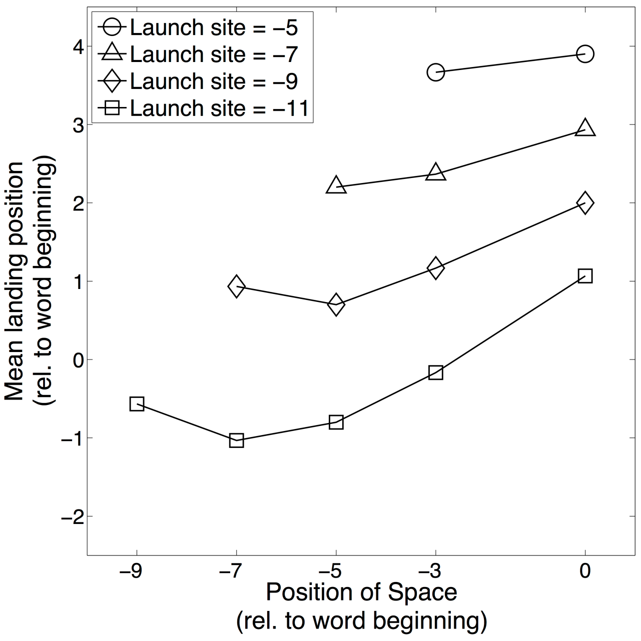

Figure S1. Initial mean landing positions as a function of the position of the first space to the right of the launch site.

References

Pollatsek, A., & Rayner, K. (1982). Eye movement control in reading: The role of word boundaries. Journal of Experimental Psychology. *Human Perception and Performance, 8*, 817–833. doi:10.1037/0096-1523.8.6.817

Rayner, K., & Pollatsek, A. (1996). Reading unspaced text is not easy: Comments on the implications of Epelboim et al.'s (1994) study for models of eye movement control in reading. *Vision Research, 36*, 461–470.
